# Pyogenic granuloma and nodular Kaposi’s sarcoma: dermoscopic clues for the differential diagnosis

**DOI:** 10.3906/sag-1902-60

**Published:** 2019-10-24

**Authors:** Ömer Faruk ELMAS, Necmettin AKDENİZ, Emine Müge ACAR, Asuman KİLİTÇİ

**Affiliations:** 1 Department of Dermatology and Venereology, Faculty of Medicine, Ahi Evran University, Kırşehir2 Turkey; 2 Department of Dermatology and Venereology, Faculty of Medicine, İstanbul Medeniyet University, İstanbul Turkey; 3 Department of Dermatology and Venereology, Faculty of Medicine, Ahi Evran University, Kırşehir Turkey; 4 Department of Pathology, Faculty of Medicine, Ahi Evran University, Kırşehir Turkey

**Keywords:** Dermoscopy, Kaposi’s sarcoma, pyogenic granuloma

## Abstract

**Background/aim:**

Pyogenic granuloma (PG)-like nodular Kaposi’s sarcoma (KS) has been previously demonstrated in several studies. However, to the best of our knowledge, no original study investigating the dermoscopic differential diagnosis of PG and KS exists in the relevant literature. In this study we aimed to identify dermoscopic findings providing useful clues to differential diagnosis between the two entities.

**Materials and methods:**

Patients with histopathologically confirmed PG or nodular KS were included in the study. Demographic, clinical, dermoscopic, and histopathological findings of the cases were retrospectively reviewed.

**Results:**

The most common finding observed in PG was red structureless areas (80.00%), followed by intersecting thick white lines (56.66%), ulceration (36.66%), and collarette scale (33.33%). The most common findings detected in nodular KS were polychromatic structures (56.66%) and red (46.66%) and white (13.33%) structureless areas, respectively.

**Conclusion:**

Intersecting thick white lines seem to be the strongest dermoscopic clue to PG. Striate surface scaling (n = 6) was a novel finding identified for PG. Here we also described a new vascular pattern (widespread vessels composing a network) for nodular KS.

## 1. Introduction

Pyogenic granuloma (PG), also known as lobular capillary hemangioma, is a benign vascular proliferation of the skin and mucous membranes. PG is usually characterized by a solitary pink to red dome-shaped nodule. The exact etiopathogenesis of the disease is not clearly known [1].

Kaposi’s sarcoma (KS) is a low-grade vascular tumor associated with human herpesvirus 8 (HHV8) infection [2]. PG and the nodular form of KS may share similar clinical characteristics. The differential diagnosis of these two entities is essential as both of them differ in prognosis and necessitate different management. Histopathological examination still remains the gold standard in this respect [3].

PG-like KS has been previously demonstrated in several studies [3]. However, to the best of our knowledge, no study investigating the dermoscopic differential diagnosis of PG and KS exists in the literature. Here we aimed to

## 2. Materials and methods

### 2.1. Patients

The study was conducted in a tertiary center. Patients with a histopathological diagnosis of PG or nodular KS between January 2017 and November 2018 were included. Demographic, clinical, dermoscopic, and histopathological findings of all the patients were retrospectively reviewed.

### 2.2. Dermoscopic assessment

A thorough dermoscopic examination was performed for all the lesions included and the findings were recorded. The dermoscopic findings observed were described using Kittlerian terminology. Dermoscopic examination was performed with a polarized handheld dermoscope with 10× magnification (Dermlite 4, 3GEN Inc., San Juan Capistrano, CA, USA). Capture of dermoscopic images was performed using a high-resolution mobile camera phone attached to the dermoscope (iPhone 7 Plus, Apple Inc., Cupertino, CA, USA).

### 2.3. Inclusion and exclusion criteria

**Figure 1:**
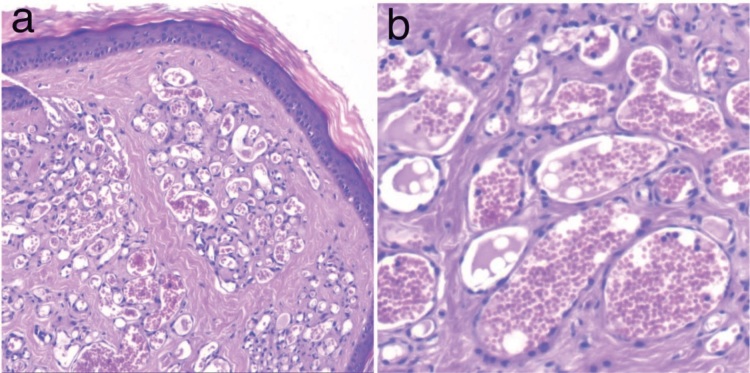
a) Vascular proliferation in a lobular fashion and fibrous septations in PG (H&E, 10×). b) Mature vascular proliferation is clearly visible (H&E, 40×).

The diagnoses of PG and nodular KS were made based on clinical and pathological correlations for all the patients. The histopathological criteria for PG were as follows: presence of vascular proliferation in a lobular fashion, fibrous septations, inflammation, and edema resembling granulation tissue with no or rare extravasated red blood cells (Figure 1).

**Figure 2:**
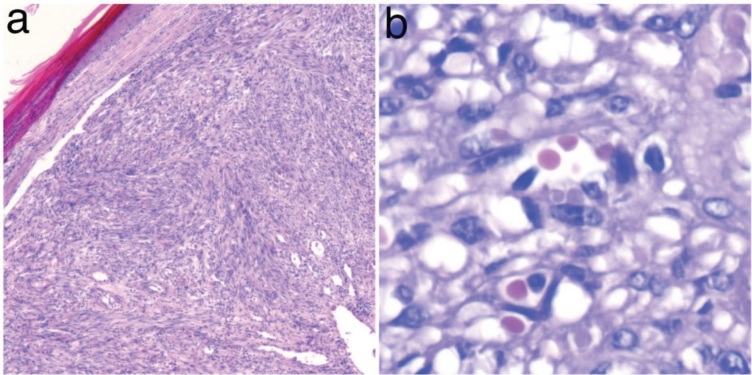
a) Spindle cell proliferation with slit-like vascular spaces in KS (H&E, 10×). b) Hyaline globules are clearly visible (periodic acid–Schiff, 40×).

The histopathological criteria for nodular KS were as follows: presence of spindle cell proliferation forming relatively circumscribed nodules, slit-like vascular spaces, and lack of remarkable nuclear atypia. Lesions showing negative HHV8 staining were excluded from the study (Figure 2).

Patients with a history of topical or systemic treatment were excluded. The presence of histological findings indicating another skin disorder was also an exclusion criterion.

### 2.4. Statistical analysis

The relationship between two categorical independent variables was evaluated using the chi-square test. Descriptive statistics for numeric variables were represented as mean ± standard deviation, and for categorical variables as numbers and % values. SPSS 24.0 for Windows (IBM Corp., Armonk, NY, USA) was used for statistical analysis and P < 0.05 was considered as statistically significant.

### 2.5. Ethical approval

All the procedures followed the Helsinki Declaration and the study was approved by the local clinical research ethics committee (Number: 2018-24/193; Date: 25.12.2018)

## 3. Results

A total of 30 lesions of PG from 30 different patients and 30 lesions of KS from 11 different patients were enrolled in the study.

### 3.1. Pyogenic granuloma

The mean age of the patients was 20.43 ± 7.75 years; there were 16 (53.33%) women and 14 (46.66) men. The most commonly affected sites were fingers (n = 14, 46.66%). Toes (n = 4, 13.33%), palms (n = 4, 13.33%), soles (n = 3, 10.00%), the neck (n = 3, 10.00%), and the lower lip (n = 2, 6.66%) were the other localizations. The mean disease duration was 25.11 ± 10.81 days.

The most common dermoscopic findings observed were red structureless areas (n = 24, 80.00%), followed by intersecting thick white lines (n = 17, 56.66%), ulceration (n = 11, 36.66%), and collarette scale (n = 10, 33.33%). All the dermoscopic findings detected are shown in the Table.

**Table:** Distribution of dermoscopic findings observed in nodular KS and PG.

Dermoscopic features	Nodular KS(n = 30)	PG(n = 30)	P-values
Thick yellow scale	n = 2 (6.66%)	n = 5 (16.66%)	P > 0.05
Collarette scale	n = 3 (6.66 %)	n = 10 (33.33%)	P < 0.05
Irregular surface scaling	n = 1 (3.33 %)	n = 8 (26.66%)	P< 0.05
Striate surface scaling	-	n = 6 (20.00%)	P< 0.05
Red structureless	n = 14 (46.66%)	n = 24 (80.00%)	P < 0.05
White structureless	n = 4 (13.33%)	n = 6 (20.00%)	P > 0.05
Purple structureless	n = 1 (3.33%)	-	P > 0.05
Rainbow pattern	n = 17 (56.66%)	n = 5 (16.66%)	P < 0.05
Purple clods	-	n = 3 (10.00%)	P > 0.05
Red clods	-	n = 3 (10.00%)	P > 0.05
Intersecting thick white lines	-	n = 17 (56.66%)	P < 0.05
Polymorphous vascular pattern	-	n = 3 (10.00%)	P > 0.05
Irregular linear vessels	n = 1 (3.33%)	n = 4 (13.33%)	P > 0.05
Widespread vessels composing a network	n = 3 (10.00%)	-	P > 0.05
Ulceration	-	n = 11 (36.66%)	P < 0.05
Blood spots	n = 3 (10.00%)	n = 10 (33.33%)	P < 0.05
Hemorrhagic black crust	n = 3 (10.00%)	n = 7 (23.33%)	P < 0.05

### 3.2. Nodular KS

The mean age of the patients was 68.45 ± 11.01 years and the majority were male (n = 9, 81.81%). Six patients had a single lesion, followed by one with eight lesions, one with seven lesions, one with four lesions, one with three lesions, and one with two lesions. The most common localization of the lesions was a lower extremity (n = 18, 60%), followed by an upper extremity (n = 9, 30%) and the head-neck region (n = 3, 10%). None of the patients showed HIV positivity, immunosuppression, or any other malignancy.

The most common dermoscopic findings were polychromatic structures (n = 17, 56.66%), also known as “rainbow patterns”. Red (n = 14, 46.66%) and white structureless areas (n = 4, 13.33%) were the other frequent findings. All of the dermoscopic findings observed are shown in the Table.

## 4. Discussion

**Figure 3:**
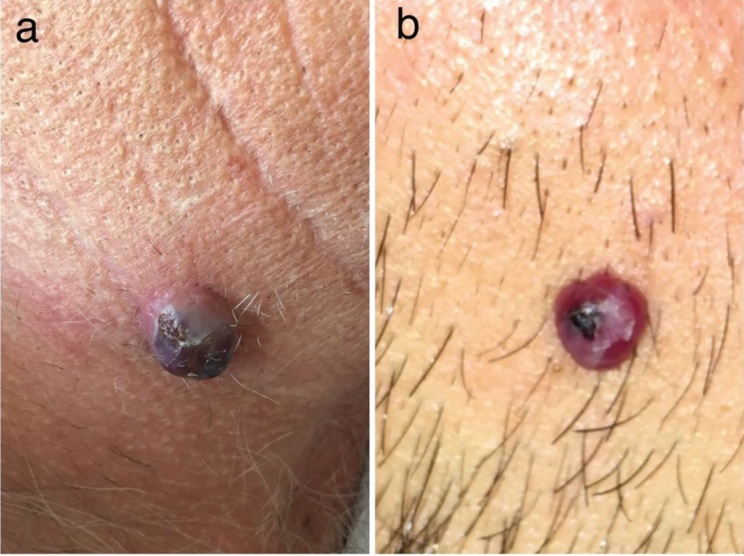
a) A solitary nodular KS localized on the neck. b) PG localized on the cheek.

PG is a benign vascular lesion mainly affecting the fingers, but it can affect all parts of the skin and mucous membranes. PG can usually be correctly diagnosed with history and the clinical appearance of the lesion [1]. However, it can also easily be misdiagnosed due to its nonspecific clinical morphology. Amelanotic melanoma, cutaneous squamous cell carcinoma, and even metastatic carcinomas are known imitators of PG [4–8]. Nodular KS (Figure 3a) and PG (Figure 3b) may also share similar clinical appearances [3]. Nodular KS usually shows numerous dome-shaped nodular lesions, unlike PG, which often presents with a single lesion [2]. However, it should be kept in mind that KS may present with a single lesion and PG may show multiple nodules [9,10].

**Figure 4:**
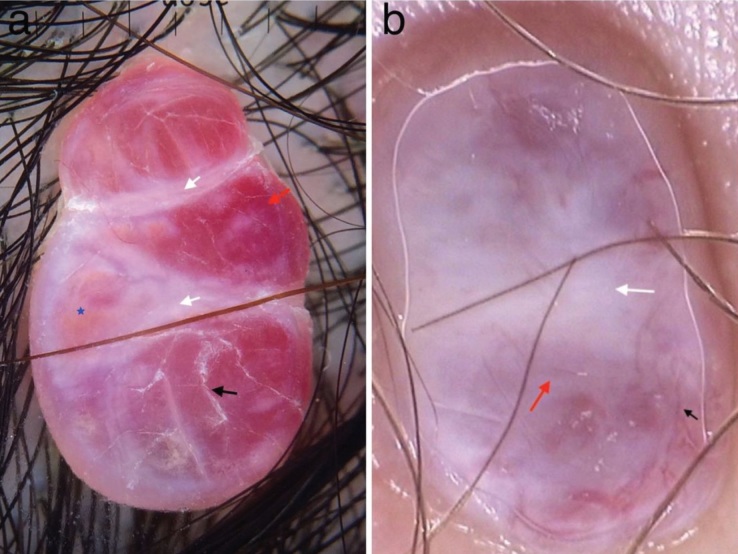
a) Red structureless areas (red arrow) intersected with thick white lines (white arrow), striated scaling (black arrow), and rainbow pattern (star) in a pyogenic granuloma localized on the scalp. b) Red (red arrow) and white (white arrow) structureless areas, irregular linear vessels (black arrows) in nodular KS.

In this study, the most common dermoscopic findings for PG were reddish structureless (homogeneous) areas (Figure 4a) (n = 24, P < 0.05). A reddish structureless area was also the most common dermoscopic feature of PG in the study of Zaballos et al. [4]. We also observed that 14 of the lesions with KS showed red structureless areas (Figure 4b). “Bluish-reddish coloration” is a previously described dermoscopic finding for KS [11]. The red structureless area is not considered as a specific clue to vascular tumors. It can be seen in many cutaneous conditions, including amelanotic melanoma and spitz nevus. The histological counterpart of the red structureless area in PG and KS is thought to be numerous capillary proliferations in the dermis [12]. White intersecting lines, also known as white rails (Figure 4a), can be described as whitish bands intersecting the lesion [4]. White intersecting lines (n = 17, 56.66%, P < 0.05) were the second most frequent dermoscopic finding of PG in the present study. None of the lesions with KS showed this finding. In the study of Zaballos et al., white intersecting lines were found in 74% of the lesions in PG cases [4]. White intersecting lines are not specific to PG and they can also be seen in melanoma and basal cell carcinoma [4]. White intersecting lines observed in PG reflect fibrous septations between lobular proliferation of mature vascular structures [13].

**Figure 5:**
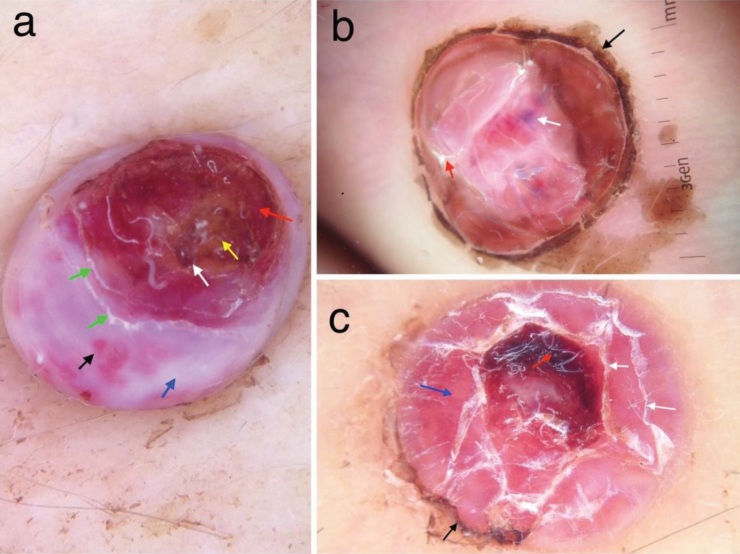
a) Ulceration (red arrow), thick yellow scale (yellow arrow), blood spots (white arrow), striated scaling (green arrows), white structureless area (blue arrow), red globules (black arrow) in PG. b) Peripheral collarette (black arrow), irregular scaling (red arrow), and rainbow pattern (white arrow) in PG. c) Red structureless (blue arrow), peripheral collarette (black arrow), blood spots (red arrow), and striated scaling (white arrows) in PG.

White structureless areas (Figures 4b and 5a) were another finding that we observed in both KS and PG. We think that this finding reflects the presence of broader fibrous tissue.

Ulceration (Figure 5a), hemorrhagic crusts (Figure 5a), and blood spots (Figure 5a) are common findings of PG as it may bleed easily [4]. Ulceration (n = 11, 36.66%), blood spots (n = 10, 33.33%), and hemorrhagic crusts (n = 7, 23.33%) were also observed in dermoscopic examination of the lesions in cases of PG. Only three KS lesions showed blood spots and crust formation. None of the KS lesions showed ulceration or crusts.

**Figure 6:**
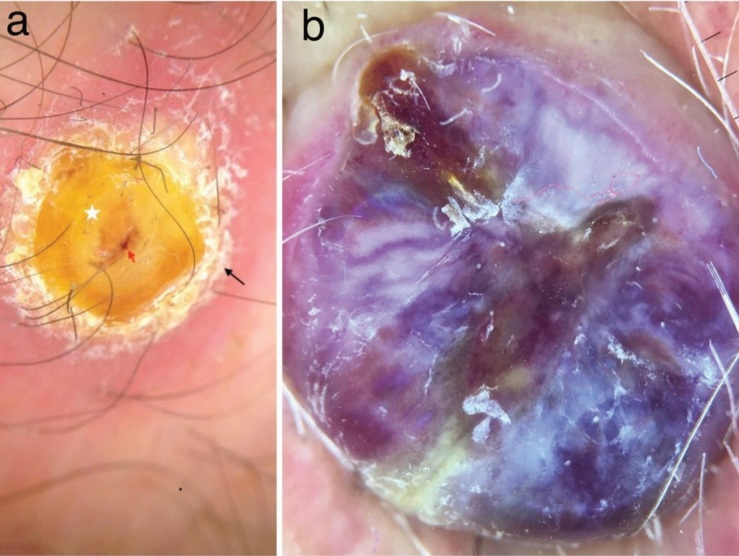
a) Peripheral collarette (black arrow), blood spots (red arrow), and thick yellow scale (star) in nodular KS. b) Rainbow pattern in nodular KS.

Peripheral collarette scale is an arcuate squamous structure surrounding the lesion [4]. This finding was found in 10 (33.33%) PG lesions (Figure 5b) and 3 (6.66%) KS lesions (Figure 6a). The histological counterpart of collarette scale is adnexal epidermal acanthosis [13]. Peripheral collarette is not a specific sign of PG or KS. It can also be seen in melanoma, angiokeratoma, and basal cell carcinoma [4]. We also observed fine striated surface scaling (Figures 5a and 5c) in 6 PG lesions while it was not detected in KS at all. To the best of our knowledge, striated surface scaling was not described for PG previously.

Polychromatic structures, also known as rainbow patterns, are described as the presence of many different colors juxtaposed next to each other on polarized dermoscopy [14]. A rainbow pattern was first described as a specific clue to KS but it has subsequently been observed in many conditions including blue nevus, angiokeratoma, hypertrophic scars, stasis dermatitis, and acroangiodermatitis [14,15]. However, to our knowledge, there is no another study identifying this pattern in PG. The pattern does not have a distinct histological correlation but it may reflect a vascular lumen-rich pattern of closely arranged “back-to-back” vascular structures [16]. Vázquez- López et al. suggested the term “dichroism” for the complex interaction of the lesional skin and polarized light. According to them, light in different statuses of polarization is absorbed in different amounts as it penetrates into an object. The heterogeneous and layered nature of the dermis determines the absorbance and retardance of polarized light, resulting in a spectrum of colors [17]. The pattern was observed in 56.66% of the KS lesions (Figure 6b) and 16.66% of the PG lesions (Figure 4a). However, the rainbow pattern observed in PG was subtle, unlike those of KS.

**Figure 7:**
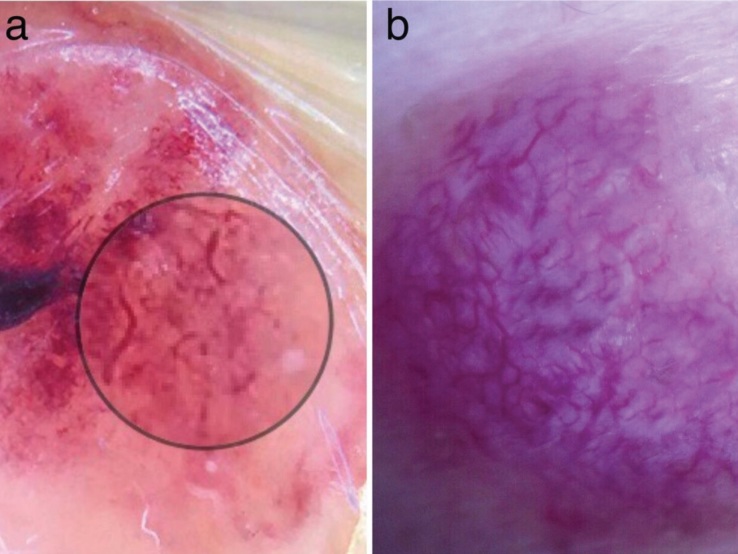
a) Polymorphous vascular pattern including irregular linear, coiled, and dotted vessels in PG. b) Widespread linear vessels composing a network in nodular KS.

When it comes to the vascular structures, 7 (23.33%) of the PG lesions (Figure 7a) and 4 (13.33%) of the KS lesions (Figure 7b) showed the presence of vascular structures. Distribution of the types of vascular structures is detailed in the Table. In the study of Zaballos et al., 45% of the PG lesions showed vascular structures [4]. We observed that 3 of the KS lesions showed widespread vessels composing a network (Figure 7b), which had not been described for KS previously.

The role of immunosuppression is well known in the HIV-associated type of KS. However, HIV is usually negative in the classical type of KS [18]. In the present study, all of the patients had classical KS and HIV positivity was not detected at all.

In conclusion, the presence of reddish structureless areas along with intersecting thick white lines seems to be a strong clue to PG. Polychromatic structures were the main dermoscopic findings of KS; however, 5 of the PG lesions also showed this pattern in a subtle manner. Here we identified a novel vascular pattern (widespread vessels composing a network) for KS, which we observed in 3 KS lesions. Striate surface scaling was another novel finding that we identified for PG. To our knowledge, this is the only original study focusing on the dermoscopic differentiation of PG and KS.
